# Fecal microbial determinants of fecal and systemic estrogens and estrogen metabolites: a cross-sectional study

**DOI:** 10.1186/1479-5876-10-253

**Published:** 2012-12-21

**Authors:** Roberto Flores, Jianxin Shi, Barbara Fuhrman, Xia Xu, Timothy D Veenstra, Mitchell H Gail, Pawel Gajer, Jacques Ravel, James J Goedert

**Affiliations:** 1 Infections and Immunoepidemiology Branch, Division of Cancer Epidemiology and Genetics, National Cancer Institute, 6120 Executive Boulevard, Room 7068, Rockville, MD 20852, USA; 2 Cancer Prevention Fellowship Program, National Cancer Institute, Rockville, MD, 20852, USA; 3 Biostatistics Branch, Division of Cancer Epidemiology and Genetics, National Cancer Institute, Rockville, MD, 20852, USA; 4 Hormonal and Reproductive Epidemiology Branch, Division of Cancer Epidemiology and Genetics, National Cancer Institute, Rockville, MD, 20852, USA; 5 Laboratory of Proteomics and Analytical Technologies, Advanced Technology Program, SAIC-Frederick, Frederick, MD, 21702, USA; 6 Institute of Genome Sciences, University of Maryland School of Medicine, Baltimore, MD, 21201, USA

**Keywords:** Microbiome, Feces, Enterohepatic circulation, β-glucuronidase, β-glucosidase, Postmenopausal estrogens, Fecal estrogens, Estrogen metabolites

## Abstract

**Background:**

High systemic estrogen levels contribute to breast cancer risk for postmenopausal women, whereas low levels contribute to osteoporosis risk. Except for obesity, determinants of non-ovarian systemic estrogen levels are undefined. We sought to identify members and functions of the intestinal microbial community associated with estrogen levels via enterohepatic recirculation.

**Methods:**

Fifty-one epidemiologists at the National Institutes of Health, including 25 men, 7 postmenopausal women, and 19 premenopausal women, provided urine and aliquots of feces, using methods proven to yield accurate and reproducible results. Estradiol, estrone, 13 estrogen metabolites (EM), and their sum (total estrogens) were quantified in urine and feces by liquid chromatography/tandem mass spectrometry. In feces, β-glucuronidase and β-glucosidase activities were determined by realtime kinetics, and microbiome diversity and taxonomy were estimated by pyrosequencing 16S rRNA amplicons. Pearson correlations were computed for each log_e_ estrogen level, log_e_ enzymatic activity level, and microbiome alpha diversity estimate. For the 55 taxa with mean relative abundance of at least 0.1%, ordinal levels were created [zero, low (below median of detected sequences), high] and compared to log_e_ estrogens, β-glucuronidase and β-glucosidase enzymatic activity levels by linear regression. Significance was based on two-sided tests with α=0.05.

**Results:**

In men and postmenopausal women, levels of total urinary estrogens (as well as most individual EM) were very strongly and directly associated with all measures of fecal microbiome richness and alpha diversity (R≥0.50, *P*≤0.003). These non-ovarian systemic estrogens also were strongly and significantly associated with fecal *Clostridia* taxa, including non-*Clostridiales* and three genera in the *Ruminococcaceae* family (R=0.57−0.70, *P*=0.03−0.002). Estrone, but not other EM, in urine correlated significantly with functional activity of fecal β-glucuronidase (R=0.36, *P*=0.04). In contrast, fecal β-glucuronidase correlated inversely with fecal total estrogens, both conjugated and deconjugated (R≤-0.47, *P*≤0.01). Premenopausal female estrogen levels, which were collected across menstrual cycles and thus highly variable, were completely unrelated to fecal microbiome and enzyme parameters (*P*≥0.6).

**Conclusions:**

Intestinal microbial richness and functions, including but not limited to β-glucuronidase, influence levels of non-ovarian estrogens via enterohepatic circulation. Thus, the gut microbial community likely affects the risk for estrogen-related conditions in older adults. Understanding how *Clostridia* taxa relate to systemic estrogens may identify targets for interventions.

**Trial registration:**

Not applicable.

## Background

Breast cancer risk is increased among postmenopausal women who have high levels of endogenous estrogens [1-3]. Conversely, high estrogen levels are associated with a reduced risk of osteoporosis and hip fracture in both postmenopausal women and elderly men [4-6]. Metabolism of estrogens occurs predominantly in the liver, including hydroxylation and conjugation [7]. Conjugated estrogens are excreted in the bile and ultimately pass into the distal gut, where they are variably deconjugated. These liberated, biologically active hormones are reabsorbed through the mucosa and enter the circulation through the portal vein [8]. A working hypothesis is that this enterohepatic recirculation affects the half-life and systemic levels of estrogens in men and postmenopausal women, and perhaps during the luteal phase in premenopausal women [9-11]. Except for obesity [3,12], determinants of non-ovarian estrogen levels remain enigmatic [13,14]. The diversity of the gut microbiota, which is inversely associated with body mass index (BMI) and obesity [15,16], could influence systemic estrogen levels through enzymatic and other pathways [11].

To characterize the distal gut microbiota and its associations with phenotypes, we developed methods that provide highly reproducible, DNA pyrosequencing-based classification of the diversity and major phyla of the fecal microbiome [16], functional activities of two fecal microbial deconjugating enzymes (β-glucuronidase and β-glucosidase) [17], and associations of these with each other and with broad characteristics of human volunteers [16-18]. Herein, using liquid chromatography/tandem mass spectrometry (LC-MS/MS) for highly sensitive and reproducible detection of the parent estrogens (estrone and estradiol) and 13 estrogen metabolites (EM) [19], we investigated the following: 1) whether systemic estrogen levels were associated with fecal microbial diversity or particular taxa; 2) whether systemic estrogen levels were associated with fecal β-glucuronidase and β-glucosidase activities; 3) whether systemic estrogen levels were associated with fecal estrogen levels (both deconjugated and conjugated); and 4) whether fecal estrogen levels were associated with fecal microbial diversity.

## Methods

The study design and analyses were consistent with the Strengthening the Reporting of Observational studies in Epidemiology – Molecular Epidemiology (STROBE-ME) statement [20].

### Participants

Following review and approval of the protocol by the National Cancer Institute (NCI) Special Studies Institutional Review Board, healthy volunteer employees of the NCI Division of Cancer Epidemiology and Genetics were recruited to assess the reproducibility of microbial measures in self-collected fecal specimens and associations with urine estrogens. Following face-to-face instructions and informed consent, participants were provided a toilet-attached pouch (Protocult, Rochester, MN), from which they collected aliquots of an early or mid-morning stool, as well as a simultaneous urine specimen. After specimen collection, they completed a brief self-administered questionnaire on demographics, broad dietary categories, ease-of-use of two different fecal collection devices, and factors potentially related to gut microbiota, specifically age, sex, height, waist size, current weight, weight change within 12 months, inflammatory bowel disease, gastrointestinal surgery, cancer and other serious disease, food allergy, and dietary restrictions (vegan or vegetarian, gluten, lactose, peanuts, pork or shellfish) [16]. Postmenopausal women were those over age 50 years with no menses, pregnancy or childbirth within the previous 12 months. Premenopausal women were those with menses, pregnancy or childbirth within the previous 12 months. None of the women had had a hysterectomy.

### Urine specimens

Urine (20-50 mL) was collected in a screw-top sterile container without preservative. Urine was chilled immediately on frozen gel packs (4°C); frozen in liquid nitrogen within 3 hours; then thawed once to make 1ml aliquots, which were re-frozen and kept at -80°C until use for analysis of estrogens and estrogen metabolites.

### Fecal specimens

Participants collected 16 aliquots, half in RNAlater (QIAGEN Inc., Valencia, CA) and half in sterile phosphate buffer saline (PBS), from various parts of a single stool. As with the urine, all fecal aliquots were chilled immediately on frozen gel packs (4°C) and frozen in liquid nitrogen within 3 hours. The fecal aliquots were stored at -80°C until used for DNA and protein extraction.

### Protein extraction

Approximately 0.5gr of thawed feces was transferred onto a 10 mL conical tube containing 5 mL of extraction buffer (60 mM Na_2_HPO_4_, 40 mM NaH_2_PO_4_, 10 mM KCl, 1 mM MgSO_4_) and kept on ice. Fecal material was homogenized by heavy vortexing for 1 min and bacterial cells were lysed by sonication using a Misonix XL2000 Ultrasonic Homogenizer (Fisher Scientific, Pittsburgh, PA) at max power for three 30-second intervals on an ice bath. Lysates were centrifuged at 21K ×g (15K rpm) for 30 minutes at 4°C using an Eppendorf 5424 microcentrifuge. Supernatant containing extracted proteins was transferred to new tubes and used to measure protein concentration and enzymatic activity. Protein concentration was estimated from each lysate using the bicinchoninic acid method according to the manufacturer’s instructions (PIERCE, Rockford, IL).

### Enzymatic activities

Protein extraction and enzymatic activities were performed as described by Goldin and colleagues [21] with slight modifications to optimize detection of the enzymatic activities. Activities of β-glucuronidase and β-glucosidase (the control enzyme) were measured in a 96-microplate format using approximately 100mg of input protein from fecal lysates (in 100 μL volume with PBS). The final reaction volume was 200 μL/well, composed of 100 μL sample and 100 μL of either 10 mM 4-Nitrophenyl-β-D-glucuronide pH7.0 (for β-glucuronidase) or 10 mM 4-Nitrophenyl-β-D-glucopyranoside pH7.0 (for β-glucosidase) preincubated at 37°C, which was added immediately before starting the enzymatic reaction. Enzymatic activity was measured in triplicates by following real-time kinetics at 37°C of the product 4-nitrophenol. The increment of the product was monitored at 405 nm for 60 minutes for fecal extracts with sufficient protein concentration, or for 5 hours for diluted fecal samples, on a Spectramax M5 (Molecular Devices, Sunnyvale, CA). Enzymatic concentrations were determined from standards curves of pure enzymes from Sigma-Aldrich (St. Louis MO, G7646 for β-glucuronidase, G4511). This relates to β-glucosidase as controls and normalized by protein input. Enzymatic activity was reported as the mean value of triplicate runs in IU/100 mg protein.

### Estrogens in urine and feces

Liquid chromatography/tandem mass spectrometry (LC-MS/MS) was used to quantify estrogens in 1mL of urine and fecal lysate in PBS [22,23]. Parent estrogens detected included estrone and estradiol; estrogen metabolites (EM) included estriol, 2-hydroxyestrone, 2-methoxyestrone, 2-hydroxyestradiol, 2-methoxyestradiol, 2-hydroxyestrone-3-methyl ether, 4-hydroxyestrone, 4-methoxyestrone, 4-methoxyestradiol, 16α-hydroxyestrone, 17-epiestriol, 16-ketoestradiol, and 16-epiestriol. A 500 μL aliquot of urine was used in the processing including enzymatic hydrolysis with glucuronidase/sulfatase-containing buffer, extraction, derivatization, and detection with stable isotope-labeled internal standards. Estrogens were quantified against calibration curves with 1000-fold linear ranges. Each batch included masked quality control samples and standards. Urine estrogens, which were determined for all participants, were expressed as pM/mg creatinine in urine, which was measured in the same samples. Fecal estrogens per pg/100 μL fecal lysate were likewise determined for 7 postmenopausal women and 22 men. The 19 premenopausal women and 3 men (selected at random) were excluded for cost considerations. To estimate deconjugated versus conjugated estrogen levels, LC-MS/MS on the fecal lysates was repeated without enzymatic hydrolysis. Conjugated estrogen was the level without hydrolysis, and deconjugated estrogen was the level with hydrolysis minus the level without hydrolysis. The current analysis considered estradiol, estrone, the EM grouped into three major hydroxylation pathways (2-, 4- and 16-hydroxylation), and the sum of these, designated as total estrogens.

### Fecal DNA extraction

Genomic DNA from stool samples preserved and transported in RNAlater was extracted with a modification of the stool QIAamp DNA Stool mini kit (QIAGEN, Valencia, CA). Briefly, 300 mg of stool sample were mixed with 350 μL of lysis buffer composed of 0.05 M potassium phosphate buffer containing 50 μL lyzosyme (10 mg/mL), 6 μL of mutanolysin (25,000 U/ml; Sigma-Aldrich) and 3 μL of lysostaphin (4 U/mL in sodium acetate; Sigma-Aldrich). The mixture was incubated for 1 hour at 37°C then 10 μL proteinase K (20 mg/ml), 100 μL 10% SDS, 20 μL RNase A (20 mg/ml) were added, and the mixture was incubated for 1 hour at 55°C. Microbial cells were lysed by mechanical disruption (bead beating) using a FastPrep instrument (MP Biomedicals, Solon, OH) set at 6.0 m/s for 30 sec. The lysate was processed using the ZR Fecal DNA extraction kit (ZYMO Research, Irvine, CA) and according to the manufacture’s recommendation omitting the lysis steps (steps 1-3). The DNA was eluted into 100 μL of Tris EDTA (TE) buffer, pH8.0.

### 454 Pyrosequencing of 16S rRNA genes

Universal primers 27F and 338R were used for PCR amplification of the V1-V2 hypervariable regions of 16S rRNA genes. The 338R primer included a unique sequence tag to barcode each sample. The primers were as follows: 27F-5′-GCCTTGCCAGCCCGCTCAGTC**AGAGTTTGATCCTGGCTCAG**-3′ and 338R-5′-GCCTCCCTCGCGCCATCAGNNNNNNNNCAT**GCTGCCTCCCGTAGGAGT**-3′, where the underlined sequences are the 454 Life Sciences® FLX sequencing primers B and A in 27F and 338R, respectively, and the bold font denotes the universal 16S rRNA primers 27F and 338R. The 8bp barcode within primer 338R is denoted by 8 Ns. Using 96 barcoded 338R primers, the V1-V2 regions of 16S rRNA genes were amplified in 96 well microtiter plates as follows: 5.0 μL 10X PCR buffer II (Applied Biosystems, Foster City, CA), 3.0 μL MgCl_2_ (25 mM; Applied Biosystems), 2.5 μL Triton X-100 (1%), 2.0 μL deoxyribonucleoside triphosphates (10 mM), 1.0 μL each of primer 27F and 338R (20 pmol/μL each), 0.5 μL AmpliTaq DNA polymerase (5 U/μL; Applied Biosystems), and 50ng of template DNA in a total reaction volume of 50 μL. Reactions were run in a PTC-100 thermal controller (MJ Research Inc., Waltham, MA) using the following cycling parameters: 5 minutes of denaturation at 95°C, followed by 20 cycles of 30 seconds at 95°C (denaturing), 30 seconds at 56°C (annealing) and 90 seconds at 72°C (elongation), with a final extension at 72°C for 7 minutes. Non-template controls were used as negative controls for each set of barcoded primers. The presence of amplicons was confirmed by gel electrophoresis on a 2% agarose gel and staining with SYBRGreen (Applied Biosystems, Foster City, CA). Equimolar amounts (~100 ng) of the PCR amplicons were mixed in a single tube, and amplification primers and reaction buffer were removed by processing the mixture with the Agencourt AMPure XP Kit (Beckman Coulter Genomics, Danvers, MA). The purified amplicon mixtures were sequenced by 454 FLX Titanium pyrosequencing (Roche Diagnostics Corp., Indianapolis, IN) with 454 Life Sciences® primer A by the Genomics Resource Center at the Institute for Genome Sciences, University of Maryland School of Medicine using protocols recommended by the manufacturer.

### Classification of operational taxonomic units

Sequence read quality used the Institute of Genome Sciences bioinformatics pipeline that complies with standard operating procedures of the National Institutes of Health Human Microbiome Project [24]. Briefly, after trimming the primer and barcode sequences, raw sequence reads were filtered using the QIIME pipeline (http://qiime.sourceforge.net)[25] with the following criteria to optimize the quality and integrity of the data: 1) minimum and maximum read length of 300 bp and 500 bp; 2) no ambiguous base calls; 3) no homopolymeric runs longer than 8 bp; 4) average quality value >q25 within a sliding window of 50 bp; 5) 60% match to a previously determined 16S rRNA gene sequence; and 6) chimera-free using the UCHIME software (http://www.drive5.com/uchime/)[26]. Sequence reads with the same barcode were binned by sample. Operational taxonomic units (OTUs) were defined using QIIME as sequences with at least 97% identity, and sequences were classified at the genus level using the Ribosomal Database Project (RDP) naïve Bayesian classifier [27].

### Statistical analysis

Relative abundance of each OTU, alpha diversity, and beta diversity were computed using QIIME [25] for each DNA sample and then averaged on four replicates for each study participant. Alpha diversity was estimated by the Shannon index, which adjusts the number of OTUs detected for their relative abundance (proportions). Shannon index is calculated as minus the sum over OTUs of the proportion of a given OTU times the logarithm of that proportion in each sample. Beta diversity, which is a measure of separation of the phylogenetic structure of the OTUs in one participant, compared to all other participants, was estimated by unweighted Unifrac distances [25]. Taxa were classified by RDP with the Visualization and Analysis of Microbial Population Structures (VAMPS, Marine Biology Laboratories, Woods Hole, MA) pipeline. Pearson correlations were computed for each log_e_ estrogen level with each log_e_ enzymatic activity level and with each microbiome alpha diversity estimate. Two-sided *P*-values were computed. For the 55 taxa with mean relative abundance of at least 0.1%, as well as the six phyla, ordinal levels were created [zero, low (below median of detected sequences), high] and compared to log_e_ β-glucuronidase and log β-glucosidase enzymatic activity levels by linear regression. Significance was based on two-sided tests with α=0.05. Analyses were conducted using the statistical software SAS version 9.2 (SAS Institute Inc, Cary, NC) and R version 2.13.0 (http://www.r-project.org/).

### Role of the funding source

This research was supported by the Intramural Research Program of the NCI, NIH, which had no direct role in the data analysis, manuscript preparation, or decision to submit for publication.

## Results

### Urinary estrogens and estrogen metabolites by sex and menopause status

**Figure 1:**
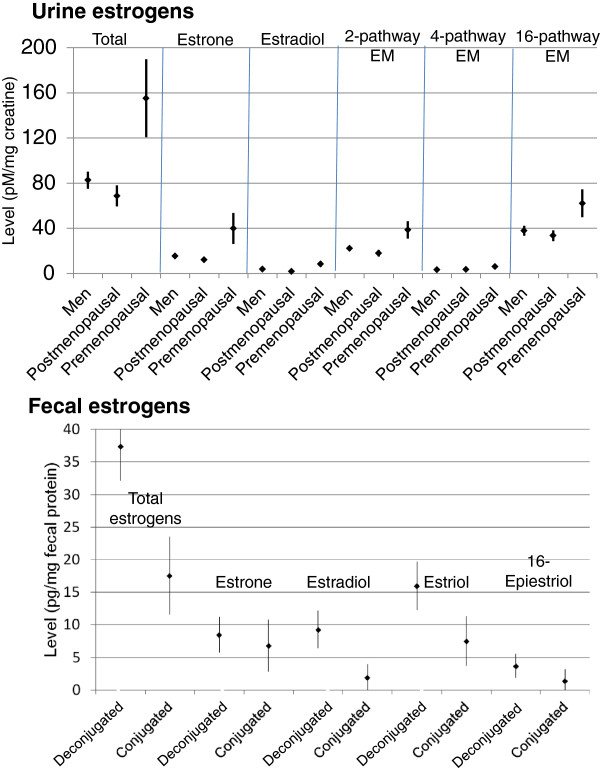
**Estrogen and estrogen metabolite (EM) levels in urine by sex and menopause status (upper panel), and in feces by conjugation status (lower panel).** Total urine estrogens were measured in 25 men, 7 postmenopausal women, and 19 premenopausal women. Fecal estrogens were measured in a combined group of 22 men and 7 postmenopausal women. Mean levels (±1 standard error) are presented.

We recruited 51 participants, including 25 men, 7 postmenopausal women, and 19 premenopausal women, who had a mean age of 40 years (range 17-65) and did not differ significantly by sex on responses on a questionnaire [16,17]. The premenopausal women included 4 in early follicular phase, 7 in mid-follicular phase, 3 in luteal phase, and 5 undefined (e.g., lactating or on contraceptive medication). The postmenopausal women ranged in age from 53 to 65 years. As reported previously [16], four participants were vegetarian; none was vegan. Thirteen participants had taken an antibiotic within six months of enrollment, including three who had taken an antibiotic within one month. A non-antibiotic prescription had been used within one month of enrollment by 17 participants and 2-6 months before enrollment by 11 additional participants. As expected [19,28-31], men and postmenopausal women had similar estrogen and EM levels in urine (Figure 1 top, and Table 1). In contrast, non-pregnant premenopausal women who were at various stages in their cycles had levels that were substantially higher and more varied. For example, mean total urine estrogen levels (pM/mg creatinine) were 82.6 [standard error of the mean (SEM) 7.6] in men, 68.7 (9.4) in postmenopausal women, and 155.1 (34.7) in premenopausal women (Figure 1 top, and Table 1).

**Table 1:** Correlation of microbial β-glucuronidase, β-glucosidase, alpha diversity, and richness with urinary estrogens and estrogen metabolites

		**Men**		**Postmenopausal women**			**Premenopausal women**	
Estrogen and estrogen metabolites (EM)*		N=25		N=7			N=19	
Total estrogens, mean (SE)		82.6 (7.6)		68.7 (9.4)			155.1 (34.7)	
-	β-glucuronidase correlation	R=0.30	*P=*0.15	R=0.31	*P=*0.51	R=0.003	*P=*0.99	
-	β-glucosidase correlation	R=0.13	*P=*0.52	R=-0.002	*P=*1.00	R=0.08	*P=*0.76	
-	Shannon index	R=0.53	*P*=0.007	R=0.71	*P*=0.07	R=0.04	*P*=0.89	
-	Observed species	R=0.59	*P*=0.002	R=0.79	*P*=0.04	R=0.12	*P*=0.63	
Estrone, mean (SE)		15.4 (2.0)		12.1 (1.6)			39.9 (13.8)	
-	β-glucuronidase correlation	R=0.45	*P=*0.03	R=0.27	*P=*0.56	R=0.03	*P=*0.89	
-	β-glucosidase correlation	R=0.32	*P=*0.12	R=-0.13	*P=*0.79	R=0.06	*P=*0.81	
-	Shannon index	R=0.35	*P=*0.08	R=0.74	*P=*0.06	R=0.005	*P=*0.98	
-	Observed species	R=0.45	*P*=0.02	R=0.80	*P*=0.03	R=0.09	*P*=0.71	
Estradiol, mean (SE)		3.8 (0.5)		1.7 (0.2)			8.4 (2.6)	
-	β-glucuronidase correlation	R=0.35	*P=*0.08	R=0.72	*P=*0.07	R=-0.06	*P=*0.81	
-	β-glucosidase correlation	R=0.29	*P=*0.17	R=0.46	*P=*0.30	R=-0.07	*P=*0.79	
-	Shannon index	R=0.25	*P=*0.22	R=0.60	*P=*0.16	R=-0.01	*P=*0.96	
-	Observed species	R=0.38	*P*=0.06	R=0.72	*P*=0.07	R=0.09	*P*=0.73	
2-pathway EM, mean (SE)		22.2 (2.1)		17.9 (2.9)			38.6 (7.7)	
-	β-glucuronidase correlation	R=0.17	*P=*0.42	R=0.13	*P=*0.79	R=0.008	*P=*0.98	
-	β-glucosidase correlation	R=0.14	*P=*0.51	R=-0.11	*P=*0.82	R=0.07	*P=*0.78	
-	Shannon index	R=0.58	*P*=0.004	R=0.47	*P*=0.29	R=0.01	*P*=0.96	
-	Observed species	R=0.64	*P*=0.0005	R=0.60	*P*=0.15	R=0.10	*P*=0.68	
4-pathway EM, mean (SE)		3.3 (0.3)		3.5 (0.6)			6.0 (1.3)	
-	β-glucuronidase correlation	R=0.06	*P=*0.76	R=0.03	*P=*0.96	R=-0.15	*P=*0.54	
-	β-glucosidase correlation	R=-0.03	*P=*0.87	R=-0.22	*P=*0.63	R=0.02	*P=*0.94	
-	Shannon index	R=0.46	*P*=0.02	R=0.26	*P*=0.57	R=-0.04	*P*=0.89	
-	Observed species	R=0.59	*P*=0.002	R=0.49	*P*=0.26	R=0.07	*P*=0.78	
16-pathway EM, mean (SE)		37.9 (4.4)		33.5 (4.8)			62.1 (12.3)	
-	β-glucuronidase correlation	R=0.22	*P=*0.30	R=0.41	*P=*0.36	R=-0.04	*P=*0.89	
-	β-glucosidase correlation	R=0.02	*P=*0.92	R=0.10	*P=*0.84	R=0.11	*P=*0.65	
-	Shannon index	R=0.43	*P*=0.04	R=0.82	*P*=0.03	R=0.06	*P*=0.82	
-	Observed species	R=0.46	*P*=0.02	R=0.84	*P*=0.02	R=0.13	*P*=0.60	

* Mean [standard error (SE)] levels of urinary estrogens and EM (grouped as 2-, 4-, or 16-pathway) in pM/mg creatinine, and log_e_-transformed for Pearson correlation and microbiome diversity analyses. Log_e_-transformed fecal microbial enzyme activity (mean of triplicate measures in duplicate Sarstedt tubes) in IU/mg protein.

### Urinary non-ovarian estrogens and fecal microbial diversity

**Figure 2:**
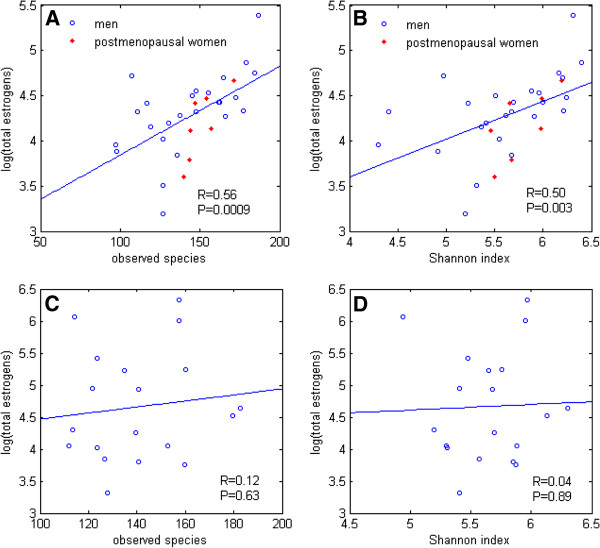
Correlations of total urine estrogen level with fecal microbiome richness (number of observed species) and alpha diversity (Shannon index) in 25 men and 7 postmenopausal women (A,B) and in 19 premenopausal women (C,D).

We focused primarily on men and postmenopausal women, grouped together, to investigate the determinants of estrogens that are primarily from non-ovarian sources. Levels of such non-ovarian estrogens were strongly associated with fecal microbiome richness and alpha diversity in 16S rRNA-based operational taxonomic units, estimated as observed species and the Shannon index, respectively (Figure 2A,B and Table 1). Adjustment for body mass index, age and sex had negligible effect on the associations of non-ovarian estrogen levels with observed species (P_adj_=0.002) and Shannon index (P_adj_=0.01). Non-ovarian estrogens were similarly associated with other alpha diversity indices (Additional file 1: Table S1). Correlations tended to be similar in men and postmenopausal women (Table 1) and for the individual estrogens and EM (Additional file 2: Figure S1).

In the premenopausal women, estrogen levels had no associations with fecal microbiome richness or alpha diversity (Figure 2C,D, Table 1, Additional file 3: Figure S2). Neither premenopausal nor non-ovarian estrogen levels were associated with beta diversity, which summarizes pairwise differences between individuals (UniFrac metrics, http://qiime.sourceforge.net)[32] (all *P*>0.20, Additional file 4: Table S2).

### Urinary non-ovarian estrogens and fecal microbial taxonomy

**Figure 3:**
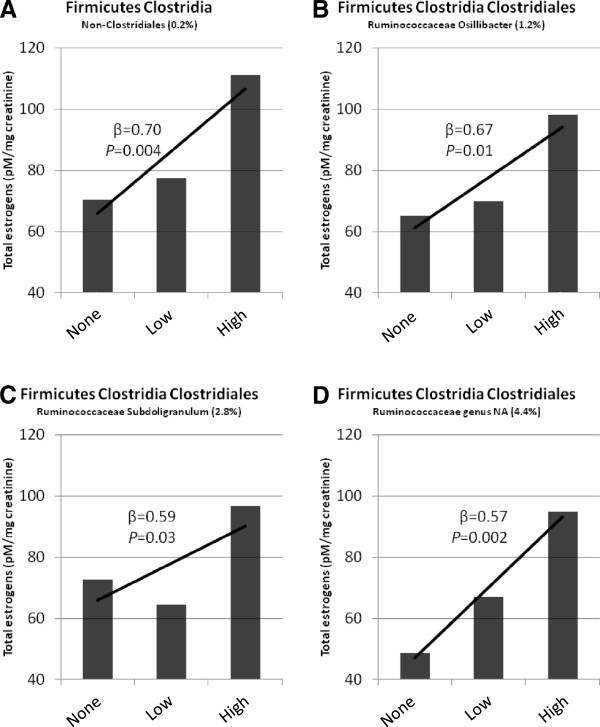
**Four *Clostridia *taxa of Firmicutes in feces significantly associated with total urine estrogens in 32 participants (25 men and 7 postmenopausal women).** For each taxon, mean relative abundance (range 0.2% - 4.4%) and levels [none, low (below median), else high relative abundance] are shown.

A few strains of bacteria have been reported to metabolize estrogens and EM *in vitro*[33], but systemic estrogen levels have not been linked to any particular organism. To explore possible taxonomic associations with estrogens, 16S rRNA pyrosequences, assigned to phylogenetic taxa with the naïve Bayesian Ribosomal Data Project (RDP) classifier [27], were compared to urine estrogen and EM levels. Estrogens were not associated with taxonomic relative abundance at the phylum level. However, of 55 taxa at the family and genus level with mean relative abundance ≥0.1%, non-ovarian urine estrogen levels were strongly and significantly associated with *Clostridia* taxa in the Firmicutes, including non-*Clostridiales* and three genera in the family *Ruminococcaceae* (β=0.57 to 0.70, *P=*0.03 to 0.002, Figure 3 and Table 2). These associations were primarily driven by levels of estrone (Table 2). Levels in premenopausal women were not associated with relative abundance at any taxonomic level. For example, the correlation of total premenopausal estrogens with abundance of non-*Clostridiales* Firmicutes was almost nil (β=-0.10, *P=*0.55).

**Table 2:** Association of bacterial phyla and selected genera with urine estrogen levels in men and postmenopausal women*

**Phyla, selected genera (mean relative abundance)**	**Totalestrogens†**		**Estrone†**		**Estradiol†**		**Estriol†**	
	**Beta†**	***P*-value**	**Beta†**	***P*-value**	**Beta†**	***P*-value**	**Beta†**	***P*-value**
Firmicutes (80.3%)	0.07	0.20	0.02	0.70	-0.0001	1.00	0.05	0.30
*Firmicutes Clostridia*								
non-*Clostridiales* (0.2%)	0.70	**0.004**	0.61	**0.01**	0.55	**0.004**	0.49	**0.02**
*Firmicutes Clostridia Clostridiales*								
*Ruminococcaceae Oscillibacter* (1.2%)	0.67	**0.01**	0.62	**0.01**	0.32	0.15	0.36	0.13
*Ruminococcaceae Subdoligranulum* (2.8%)	0.59	**0.03**	0.47	**0.04**	0.19	0.40	0.46	**0.04**
*Ruminococcaceae* genus NA (4.4%)	0.57	**0.002**	0.45	**0.01**	0.17	0.29	0.44	**0.01**
Bacteroidetes (16.9%)	-0.07	0.19	-0.02	0.67	-0.01	0.88	-0.05	0.28
Actinobacteria (1.3%)	0.0004	0.41	-0.0003	0.93	0.001	0.72	0.005	0.21
Proteobacteria (0.5%)	-0.24	0.27	-0.29	0.13	-0.28	0.10	-0.20	0.28
Fusobacteria (0.2%)	-0.19	0.30	-0.15	0.34	-0.06	0.70	-0.22	0.15
Unclassified bacteria (0.8%)	0.39	0.11	0.33	0.12	0.21	0.29	0.28	0.18

* 25 men, 7 postmenopausal women. †Level (pM/mg creatinine) in urine. †Beta values estimate the increase in log_e_ of estrogen per log_e_ copy for phyla, else per level (none, low, high) for genera and unclassified bacteria. *P*-values were not adjusted for multiple comparisons.

### Urinary non-ovarian estrogens and fecal microbial enzymes

Intestinal contents can hydrolyze several estrogens and EM [34]. These reactions have been attributed to gut luminal bacteria, based largely on the effects of antibiotics on fecal and systemic estrogen and EM levels [35]. With well validated assays on the fecal specimens from our male and postmenopausal volunteers [17], fecal β-glucuronidase activity was significantly correlated with urine estrone level (R=0.36, *P=*0.04) but not with total urine estrogens (R=0.24, *P=*0.19), estradiol (R=0.16, *P=*0.38), or EM. Activity of the control enzyme, β-glucosidase, was not related to total urine estrogens (R=0.12) or to any of the parent estrogens or EM. In pre-menopausal women, urine estrogens were not correlated with either β-glucuronidase or β-glucosidase activity (Table 1).

### Fecal estrogens, urinary estrogens, and fecal microbial enzymes

**Figure 4:**
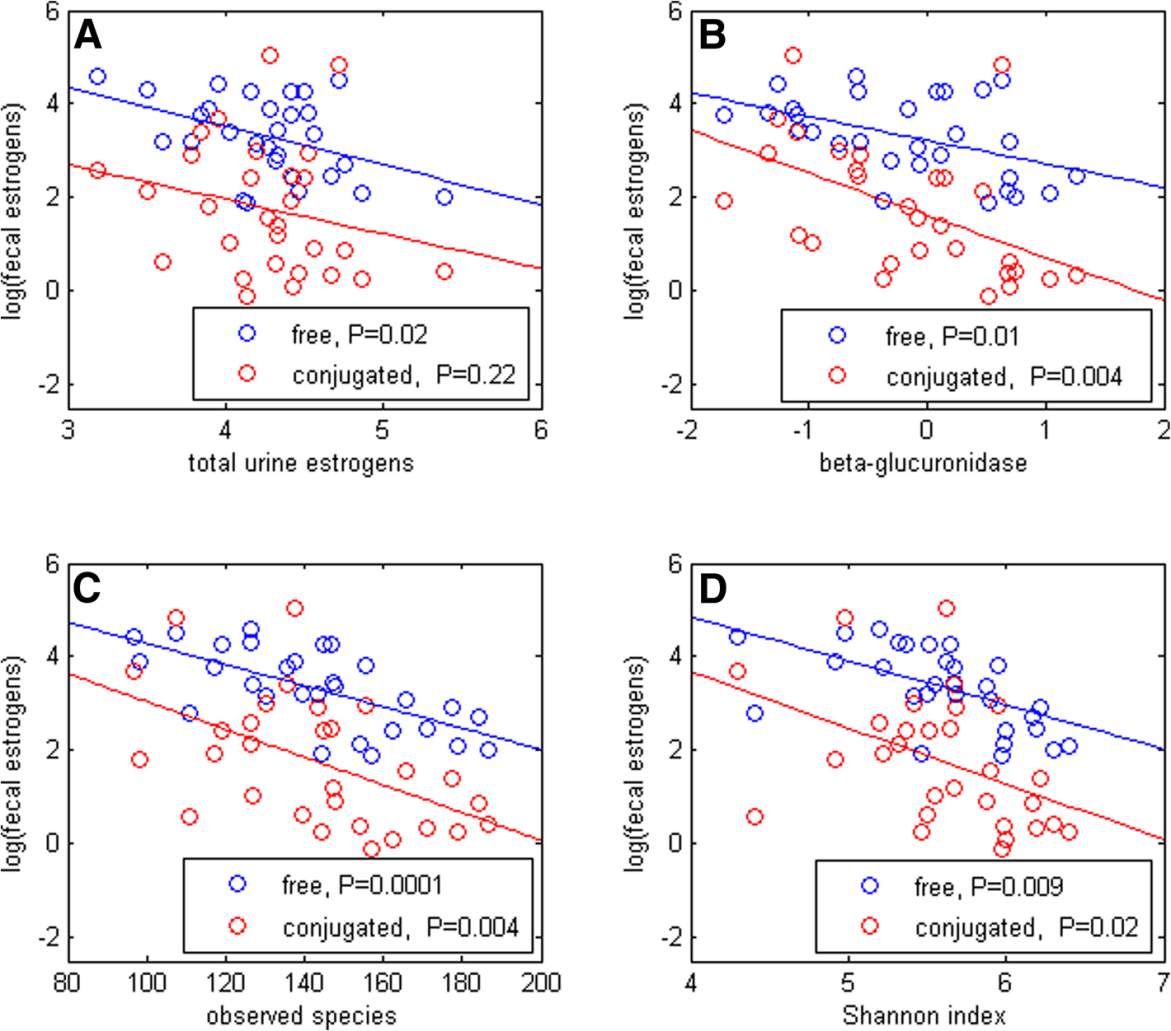
Correlations of fecal estrogen levels with urine estrogens (A), fecal β-glucuronidase activity (B), and fecal microbiome observed species and Shannon index (C,D) in 29 participants (7 postmenopausal women and 22 men).

As previously noted in a few individuals [28,34,35], in feces we readily detected both conjugated and deconjugated estrone, estradiol, estriol and 16-epiestriol (but not 11 other EM) in all 29 participants examined (Figure 1 bottom). Deconjugated fecal estrogens were inversely correlated with total estrogen levels in urine (Figure 4A, R=-0.43, *P*=0.02), and this inverse association with urine estrogens was especially strong for deconjugated fecal estrone (R=-0.50, *P*=0.005, Table 3). Conjugated estrogens and EM in feces were not significantly correlated with urinary estrogen levels (Table 3). Fecal β-glucuronidase activity was inversely correlated with both deconjugated and conjugated estrogens in feces (P≤0.01 for all except 16-epiestriol, Figure 4B). Higher fecal microbiome Shannon index and number of observed species were strongly and significantly associated with lower levels of conjugated and especially deconjugated estrogens in feces (Figure 4C,D and Table 3). The inverse correlations imply that more estrogen is excreted through feces when microbial diversity and enzymatic activity are low.

**Table 3:** Correlations of deconjugated and conjugated fecal estrogens and estrogen metabolites with total estrogen levels in urine and with β-glucuronidase activity, observed species and Shannon index in feces in 29 participants (7 postmenopausal women and 22 men)

	**Total urine estrogens**		**Beta-glucuronidase**		**Observed species**		**Shannon index**	
**Fecal estrogen**	**R**	***P*-value**	**R**	***P*-value**	**R**	***P*-value**	**R**	***P*-value**
Total, deconjugated	-0.43	0.02	-0.47	0.01	-0.66	0.0001	-0.58	0.001
Total, conjugated	-0.23	0.22	-0.52	0.004	-0.52	0.004	-0.44	0.02
Estrone, deconjugated	-0.50	0.005	-0.54	0.002	-0.66	0.00009	-0.59	0.0008
Estrone, conjugated	-0.32	0.09	-0.48	0.008	-0.44	0.02	-0.36	0.05
Estradiol, deconjugated	-0.28	0.15	-0.35	0.06	-0.65	0.0001	-0.62	0.0003
Estradiol, conjugated	-0.11	0.55	-0.37	0.05	-0.37	0.05	-0.34	0.07
Estriol, deconjugated	-0.41	0.03	-0.45	0.02	-0.55	0.002	-0.44	0.02
Estriol, conjugated	-0.22	0.25	-0.47	0.01	-0.53	0.003	-0.44	0.02
16-Epiestriol, deconjugated	-0.32	0.09	-0.28	0.15	-0.52	0.004	-0.43	0.02
16-Epistriol, conjugated	-0.01	0.95	-0.30	0.12	-0.35	0.07	-0.29	0.14

## Discussion

Our study supports, for the first time in a population of human volunteers, the longstanding theory that the intestinal microbiota affects systemic estrogen levels [9,11,28,35,36]. As postulated [11,28,35], we found that the activity of fecal β-glucuronidase (but not our control enzyme, β-glucosidase) was inversely associated with all estrogen levels in the gut, and directly associated with estrone level in the urine. More importantly, we found that the richness of the fecal microbiome (i.e., number of unique species) was very strongly and directly associated with systemic estrogens. These associations were robust to different classifications of microbiome alpha diversity, and they held for estrone, estradiol, and EM. Our beta diversity analysis suggests that estrogen levels are not associated with any particular class or cluster in the microbiome. Rather, our findings point to relatively rare taxa, particularly to certain *Clostridia* in the Firmicutes phylum. Independent replication is required, as these taxonomic associations were not specified *a priori* and may have arisen by chance because of the many taxa that were examined.

We confirmed previous reports that estrogen levels are similar in men and postmenopausal women but substantially lower and less variable than in premenopausal women [19,28-31]. In premenopausal women, ovarian estrogens dominate estrogens that are reabsorbed from the gut. Thus, our null associations in premenopausal women lend specificity to the conclusion that the intestinal microbiota affects non-ovarian systemic estrogen levels. A possible contribution of enterohepatic circulation to luteal (nadir) estrogen levels in premenopausal women would require a specially designed and controlled investigation.

The primary limitations of our study are its small size, convenience sampling and cross-sectional design. These limit the ability to detect associations and to generalize to other populations. In addition, rather than studying a disease phenotype, we merely examined estrogen levels in a random morning urine from volunteers who were highly motivated and in good health. Nonetheless, given the paucity of understanding of the determinants of systemic estrogens and of the many functions of the gut microbiota and its transient or sustained effects on the human host, our study is noteworthy for its finding that intestinal microbial richness and certain taxa may contribute to systemic estrogen levels and associated diseases. Because use of medications, including non-antibiotics [16], and elevated BMI [15,16] are inversely associated with gut microbiome alpha diversity, adjustment for these likely would have strengthened the direct associations that we found herein between alpha diversity and systemic estrogen levels. Finally, it would be useful to identify associations of microbiome parameters with better estimates of systemic estrogens, such as in 24 hour urine and repeated serum specimens.

As postulated and summarized by others [11], our results support the hypothesis that breast cancer risk in postmenopausal women and hip fracture risk in both postmenopausal women and elderly men is modulated by decades-long differences in systemic estrogens attributable to differences in the intestinal microbiota. If so, a desirable goal would be to manipulate the microbiota or specific microbial functions to reduce disease risks. For example, inhibition of fecal microbial β-glucuronidases may be possible [37], although the relatively weak associations that we found between fecal β-glucuronidase activity and urinary estrogens suggest that enzyme inhibition would not markedly reduce systemic estrogen levels. For future work, we would postulate that the gut microbiota may contribute to enterohepatic recycling via deconjugation of sulfated estrogens [38] or may mediate absorption of estrogens via inflammation, neither of which was evaluated herein. Validation of our findings in larger and more representative populations, as well as much broader and deeper understanding of how the gut contents and mucosa modulate estrogen homeostasis, are needed.

## Conclusions

Intestinal microbial richness and functions, including but not limited to β-glucuronidase, influence levels of non-ovarian estrogens via enterohepatic circulation. Thus, the gut microbial community may contribute to the risk for estrogen-related conditions in older adults. Understanding how *Clostridia* taxa relate to systemic estrogens may identify targets for interventions.

## Competing interests

The authors declare no financial or non-financial competing interests.

## Authors’ contributions

RF contributed to the protocol development; led the field work; developed, optimized, and performed the enzymatic activity assays; and led the taxonomy analyses. JS led the statistical analyses. BF led the estrogen analyses. XX developed and performed the estrogen LC-MS/MS assays. TDV supervised the LC-MS/MS development and assay performance. MHG developed the statistical analysis strategy and supervised the statistical analyses. PG performed statistical analyses of the 16S rRNA sequence data. JR development the methods for and supervised the fecal DNA extraction, 16S rRNA amplification and pyrosequencing. JJG conceived the study, obtained the funding, drafted the protocol, assisted with the field work, coordinated the analyses, and drafted the manuscript. All authors contributed to, read and approved the final manuscript.

## Supplementary Material

Additional file 1 **Table S1.** Correlation of selected measures of two measures of alpha diversity, Chao1 and phylogenetic distance whole tree indices, with levels of urinary estrogens and estrogen metabolites. Click here for file

Additional file 2 **Figure S1.** Correlation of fecal microbiome richness and alpha diversity with each parent estrogen and estrogen metabolite group in men and postmenopausal women. Click here for file

Additional file 3 **Figure S2.** Correlation of fecal microbiome richness and alpha diversity with each parent estrogen and estrogen metabolite group in premenopausal women. Click here for file

Additional file 4 **Table S2.** Association of urine estrogen levels, in a combined group of 25 men and 7 postmenopausal women, with the first five principal components of fecal microbiome beta diversity, as estimated by Unifrac. Click here for file
