# Supplementary material for: Fecal microbial determinants of fecal and systemic estrogens and estrogen metabolites: a cross-sectional study
Source: J Transl Med. 2012 Dec 21;10:253. doi: 10.1186/1479-5876-10-253 (PMC3552825; doi:10.1186/1479-5876-10-253)
Supplement: Additional file 1 — Table S1. Correlation of selected measures of two measures of alpha diversity, Chao1 and phylogenetic distance whole tree indices, with levels of urinary estrogens and estrogen metabolites. [file 1479-5876-10-253-S1.doc]

| **Supplemental Table 1.** Correlation of selected measures of two measures of alpha diversity, Chao1 and phylogenetic distance whole tree indices, with levels of urinary estrogens and estrogen metabolites. | | | | | | | |
| --- | --- | --- | --- | --- | --- | --- | --- |
|  |  |  | |  | |  | |
|  |  | **Men** | | **Postmenopausal women** | | **Premenopausal women** | |
| Estrogen and estrogen metabolites (EM)* | | N=25 | | N=7 | | N=19 | |
|  |  |  | |  | |  | |
| Total estrogens, mean (SEM) | | 82.6 (7.6) | | 68.7 (9.4) | | 155.1 (34.7) | |
| - | Chao1 | R=0.52 | *P*=0.007 | R=0.56 | *P*=0.19 | R=0.14 | *P*=0.58 |
| - | Phylogenetic distance whole tree | R=0.55 | *P*=0.005 | R=0.61 | *P*=0.15 | R=0.20 | *P*=0.41 |
|  |  |  | |  | |  | |
| Estrone, mean (SEM) | | 15.4 (2.0) | | 12.1 (1.6) | | 39.9 (13.8) | |
| - | Chao1 | R=0.39 | *P=*0.05 | R=0.73 | *P=*0.06 | R=0.11 | *P=*0.66 |
| - | Observed species | R=0.49 | *P*=0.01 | R=0.52 | *P*=0.23 | R=0.21 | *P*=0.40 |
|  |  |  | |  | |  | |
| Estradiol, mean (SEM) | | 3.8 (0.5) | | 1.7 (0.2) | | 8.4 (2.6) | |
| - | Chao1 | R=0.34 | *P=*0.10 | R=-0.16 | *P=*0.74 | R=0.09 | *P=*0.72 |
| - | Phylogenetic distance whole tree | R=0.38 | *P*=0.06 | R=0.87 | *P*=0.01 | R=0.19 | *P*=0.43 |
|  |  |  | |  | |  | |
| 2-pathway EM, mean (SEM) | | 22.2 (2.1) | | 17.9 (2.9) | | 38.6 (7.7) | |
| - | Chao1 | R=0.64 | *P*=0.0006 | R=0.48 | *P*=0.28 | R=0.12 | *P*=0.63 |
| - | Phylogenetic distance whole tree | R=0.55 | *P*=0.004 | R=0.41 | *P*=0.36 | R=0.17 | *P*=0.48 |
|  |  |  | |  | |  | |
| 4-pathway EM, mean (SEM) | | 3.3 (0.3) | | 3.5 (0.6) | | 6.0 (1.3) | |
| - | Chao1 | R=0.58 | *P*=0.003 | R=0.33 | *P*=0.46 | R=0.09 | *P*=0.72 |
| - | Phylogenetic distance whole tree | R=0.46 | *P*=0.02 | R=0.37 | *P*=0.42 | R=0.12 | *P*=0.63 |
|  |  |  | |  | |  | |
| 16-pathway EM, mean (SEM) | | 37.9 (4.4) | | 33.5 (4.8) | | 62.1 (12.3) | |
| - | Chao1 | R=0.37 | *P*=0.07 | R=0.55 | *P*=0.20 | R=0.15 | *P*=0.54 |
| - | Phylogenetic distance whole tree | R=0.41 | *P*=0.04 | R=0.70 | *P*=0.08 | R=0.20 | *P*=0.40 |
|  |  |  | |  | |  | |
|  |  |  | |  | |  | |
| * Mean [standard error (SEM)] levels in pM/mg creatinine of urinary estrogens and EM (grouped as 2-, 4-, or 16-pathway). Log-transformed fecal microbial enzyme activity (mean of triplicate measures in duplicate Sarstedt tubes) in IU/mg protein. | | | | | | | |
