# Supplementary figures and images for: Fecal microbial determinants of fecal and systemic estrogens and estrogen metabolites: a cross-sectional study
Source: J Transl Med. 2012 Dec 21;10:253. doi: 10.1186/1479-5876-10-253 (PMC3552825; doi:10.1186/1479-5876-10-253)

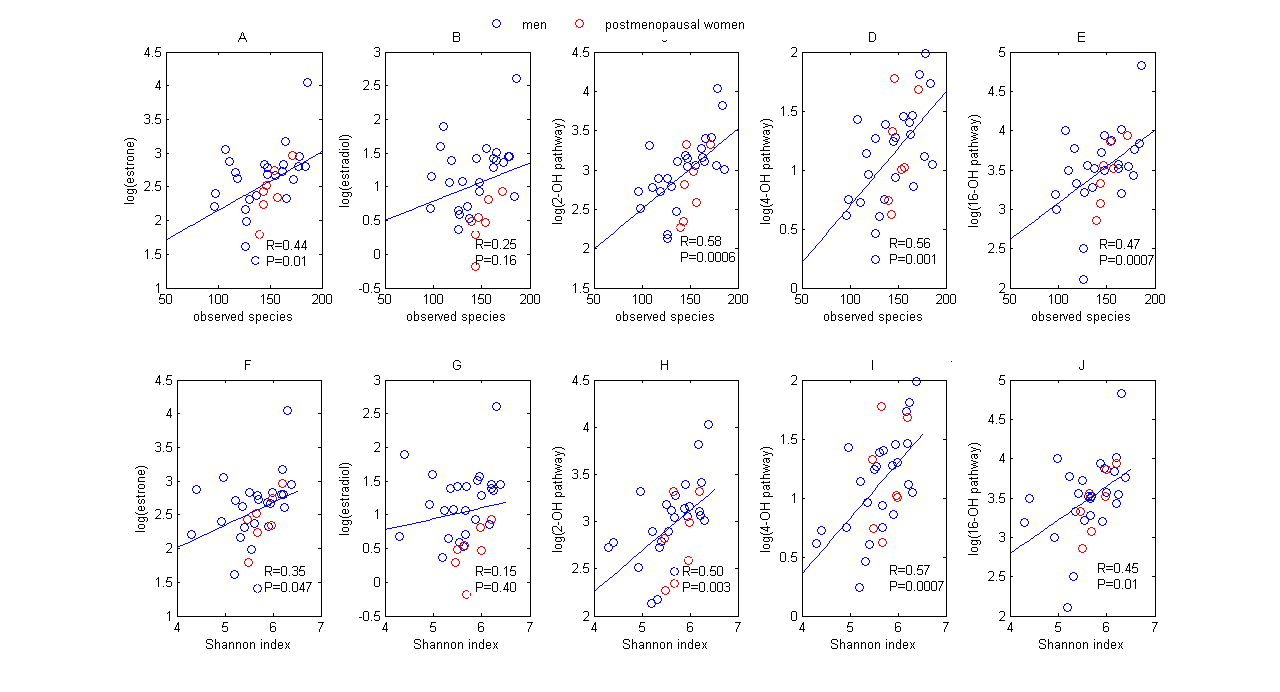

Supplement: Additional file 2 — Figure S1. Correlation of fecal microbiome richness and alpha diversity with each parent estrogen and estrogen metabolite group in men and postmenopausal women. [file 1479-5876-10-253-S2.tiff]

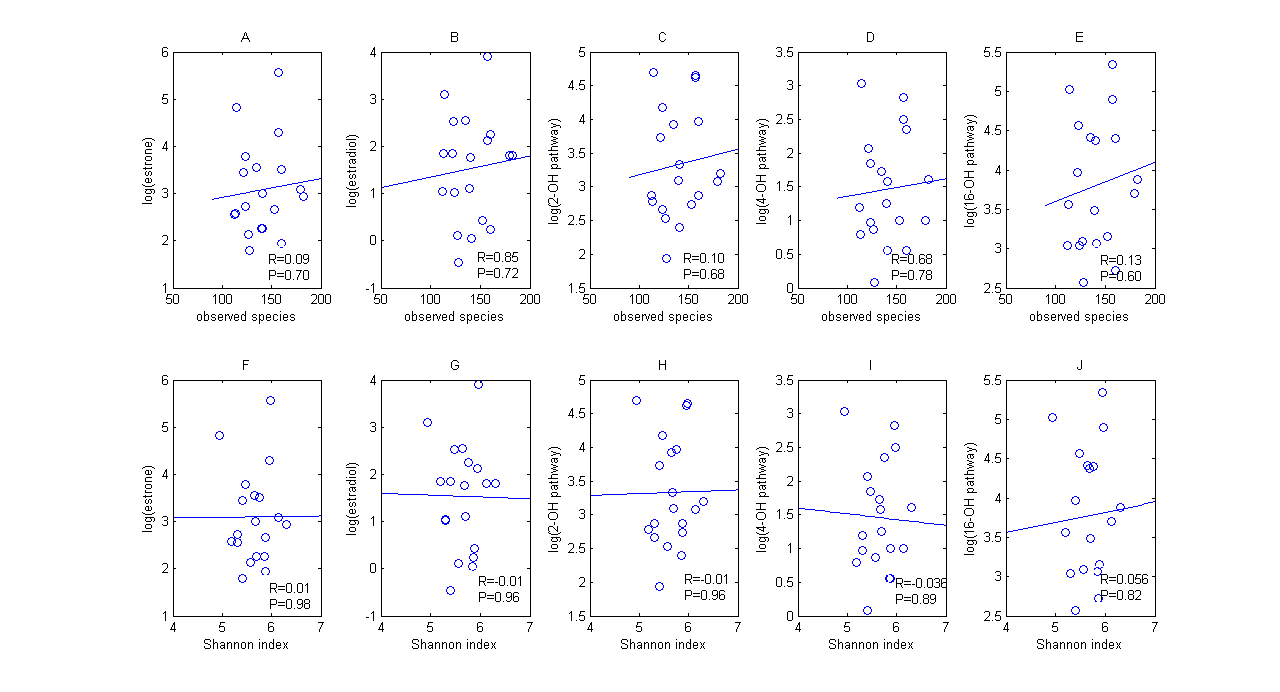

Supplement: Additional file 3 — Figure S2. Correlation of fecal microbiome richness and alpha diversity with each parent estrogen and estrogen metabolite group in premenopausal women. [file 1479-5876-10-253-S3.tiff]
