# Supplementary material for: Fecal microbial determinants of fecal and systemic estrogens and estrogen metabolites: a cross-sectional study
Source: J Transl Med. 2012 Dec 21;10:253. doi: 10.1186/1479-5876-10-253 (PMC3552825; doi:10.1186/1479-5876-10-253)
Supplement: Additional file 4 — Table S2. Association of urine estrogen levels, in a combined group of 25 men and 7 postmenopausal women, with the first five principal components of fecal microbiome beta diversity, as estimated by Unifrac. [file 1479-5876-10-253-S4.doc]

| **Supplemental Table 2.** Association of urine estrogen levels, in a combined group of 25 men and 7 postmenopausal women, with the first five principal components of fecal microbiome beta diversity, as estimated by Unifrac. | | | | | | |
| --- | --- | --- | --- | --- | --- | --- |
|  |  |  |  |  |  |  |
|  |  | pc1 | pc2 | pc3 | pc4 | pc5 |
| Total estrogens | β | -0.213 | 0.226 | 0.102 | 0.062 | 0.060 |
|  | P-value | 0.2424 | 0.2130 | 0.5798 | 0.7363 | 0.7463 |
| Estrone | β | -0.229 | 0.158 | 0.032 | 0.085 | 0.133 |
|  | P-value | 0.2082 | 0.3886 | 0.8605 | 0.6451 | 0.4668 |
| Estradiol | β | -0.179 | 0.119 | -0.203 | 0.079 | 0.132 |
|  | P-value | 0.3268 | 0.5152 | 0.2655 | 0.6683 | 0.4704 |
| 2-pathway EM | β | -0.231 | 0.179 | 0.065 | 0.056 | 0.004 |
|  | P-value | 0.2043 | 0.3270 | 0.7246 | 0.7609 | 0.9835 |
| 4-pathway EM | β | -0.205 | 0.225 | 0.115 | 0.173 | -0.130 |
|  | P-value | 0.2596 | 0.2156 | 0.5294 | 0.3449 | 0.4797 |
| 16-pathway EM | β | -0.136 | 0.230 | 0.141 | 0.070 | 0.026 |
|  | P-value | 0.4568 | 0.2053 | 0.4424 | 0.7016 | 0.8882 |
